# The Association of Unhealthy Eating Behaviors with Sleep Quality Outcomes Among University Students: A Cross-Sectional Study

**DOI:** 10.3390/nu17223580

**Published:** 2025-11-15

**Authors:** Maha Al-Jawarneh, Shalini Chauhan, Ildikó Csölle, Szimonetta Lohner

**Affiliations:** 1 Doctoral School of Health Sciences, Faculty of Health Sciences, University of Pécs, 7621 Pecs, Hungary; 2 Cochrane Hungary, Medical School, University of Pécs, 7624 Pecs, Hungary; 3 Physical Education and Exercise Centre, Medical School, University of Pécs, 7624 Pecs, Hungary

**Keywords:** sleep quality, chrono-nutrition, dietary habits, meal timing, university students

## Abstract

**Background**: This study examined the association between specific unhealthy eating behaviors and sleep quality among university students. Understanding how dietary habits affect sleep during significant lifestyle transitions associated with university experience can inform health promotion strategies. **Methods**: A cross-sectional design was employed using a self-reported questionnaire to assess eating behaviors, timing of meals, and sleep-related behavior among a sample of international university students. Sleep quality was measured using the Pittsburgh Sleep Quality Index (PSQI) tool. Statistical analyses were used to assess the relationship between eating patterns, overall sleep quality, and its components. **Results**: More than half of the students had poor sleep quality (51.7%). Daytime dysfunction was significantly more common in females than in males (27.9% vs. 8.3%, respectively; *p* < 0.001). Conversely, poor sleep efficiency was more prevalent among males than females (27.5% vs. 15.8%; *p* = 0.008). Multivariate logistic regression revealed that, compared to students who did not frequently consume heavy evening meals, those who did were more likely to experience poor sleep quality (OR = 2.73, 95% CI: 1.575–4.731). Similarly, those who frequently substitute snacks for main meals were more likely to experience poor sleep quality than those who did not (OR = 2.68, 95% CI: 1.465–4.895). Finally, students who ate within three hours of bedtime had higher odds of poor sleep quality compared to those who had their last meal more than three hours before bedtime (OR = 2.06, 95% CI: 1.173–3.629). **Conclusions**: Unhealthy dietary habits, such as consuming heavy evening meals, substituting snacks for main meals, and having a short meal-to-bedtime interval, were significantly associated with poor sleep quality. Interventions promoting healthier dietary patterns and appropriate meal timing could help improve sleep in this population.

## 1. Introduction

Poor sleep quality (SQ) is a significant public health concern, associated with increased risk of morbidity and mortality [1]. Research indicates that it negatively impacts cognitive functions, such as concentration and memory, exacerbates physical and mental health problems, and ultimately raises healthcare costs [2,3].

Sleep disturbances, including insomnia, are more prevalent in young adults, especially university students, than in the general population [4,5,6]. The high incidence of sleep problems among students might be due to the exceptional demands of university life. There is often increased academic pressure, irregular schedules for classes, and active engagement in social life [7,8,9,10]. Furthermore, standard lifestyle changes, characterized by increased caffeine consumption and screen time, may alter circadian regulation and sleep architecture [11,12]. Subsequently, these impairments lead to the development of unhealthy dietary habits and diminished sleep quality [13,14].

Sleep quality (SQ) is multidimensional and includes the individual’s subjective evaluation of their sleep, depending on factors such as sleep duration, efficiency, latency, and nighttime awakenings [15]. Many tools, such as the Pittsburgh Sleep Quality Index (PSQI), have been widely used and validated to measure these characteristics [15,16].

A growing body of research has examined the complex interactions between dietary patterns and sleep quality and duration [17]. For example, Melo et al. found that higher food consumption in the evening was associated with increased nocturnal alertness, poorer sleep efficiency, and greater apnea-hypopnea index (AHI) [18,19]. In a further investigation, participants were classified according to the timing of their food intake into early eaters, late eaters, and meal skippers. The findings showed worse sleep quality (SQ) and more daytime sleepiness in late eaters than in early eaters [20]. Choi et al. also demonstrated that eating late at night and skipping breakfast were correlated with poorer sleep quality (SQ) and a higher prevalence of obstructive sleep apnea [21]. Together, these findings highlight the significant influence of dietary timing on sleep outcomes.

Chrono-nutrition is defined by three key aspects of human dietary habits: timing, regularity, and frequency of food intake [22,23,24,25]. In 1986, Alain Delabos proposed this concept, emphasizing that eating at certain times of the day should ideally harmonize with the body’s endogenous circadian rhythms, which are regulated by a central pacemaker located in the suprachiasmatic nucleus (SCN) of the hypothalamus. This central clock harmonizes the peripheral oscillators in various metabolic organs, such as the liver, pancreas, and adipose tissues. The timing of meals acts as a powerful zeitgeber (time-giver) that enables these clocks to function optimally. When eating aligns with the circadian cycle (primarily during daylight hours), metabolic processes such as lipid metabolism and glucose regulation are optimized, helping to maintain a stable sleep–wake cycle [26].

Conversely, mistimed eating patterns desynchronize peripheral clocks from the central SCN pacemaker, which can suppress nocturnal melatonin secretion, raise core body temperature and sympathetic nervous system activity when the body is in a state preparing for rest, which impairs sleep onset and maintenance, and cause many health consequences such as increased risk of type 2 diabetes mellitus, cardiometabolic diseases, obesity, and poor sleep quality [18,19,26,27,28,29,30,31].

Due to the global population of international students having substantially increased, going from 2 million in 2000 to over 6 million in 2021, as the World Migration Report indicates, it is critical to investigate how the distinct dietary and lifestyle habits of this group can be associated with their sleep quality [32,33,34]. These habits include skipping breakfast, late-night snacking, irregular meal timing, and substituting snacks for main meals.

The aim of this study was to explore the association between detrimental dietary behaviors and sleep quality among international university students. Specifically, eating close to bedtime, substituting snacks for main meals, late-night snacking, skipping breakfast, consuming heavy evening meals, and irregular eating schedules. We hypothesized that the aforementioned dietary patterns are associated with poorer sleep quality among international university students.

## 2. Materials and Methods

### 2.1. Methodology

The research design, performance, and writing adhered to the guiding principles introduced in the Strengthening the Reporting of Observational Studies in Epidemiology (STROBE) guidelines [35].

### 2.2. Study Design

This cross-sectional quantitative study was conducted between September and November 2024 at the University of Pécs, Hungary, using a self-administered questionnaire that included the validated Pittsburgh Sleep Quality Index (PSQI).

### 2.3. Participants

All international students (approximately 5000), aged 18 years or older, and enrolled in the fall semester of the 2024/2025 academic year at the University of Pécs, were considered eligible for participation [36]. Those who did not meet these criteria were deemed ineligible. Students were invited to participate through convenience sampling, a technique selected for its practicality, cost-effectiveness, and suitability for research within relatively homogeneous populations, such as university student cohorts [37].

A total of 422 students were approached; 32 declined to participate, and 390 initially completed the survey. Following data screening, the final dataset comprised 385 complete questionnaires, while a small number of responses (*n* = 5) were excluded due to missing data (the entire PSQI section was blank). Given the small proportion and non-systematic nature of the missingness, this exclusion was not expected to introduce significant bias.

### 2.4. Data Collection

Data were collected through on-site visits to the University of Pécs campuses. Students were approached and provided with a detailed explanation of the study’s objectives. After obtaining informed consent, a paper-based questionnaires were given to the participants to complete. Participation was voluntary.

### 2.5. Sample Size

World Health Organization (WHO) guidelines for prevalence studies formula was used to determine the required sample size [38,39]:

$$n=\frac{Z^{2}\times p(1-p)}{d^{2}}$$

Based on the following parameters: a 95% confidence level, a *Z*-score of 1.96 (corresponding to the 95% confidence level), a 5% margin of error (*d*), and an assumed population proportion (*p*) of 0.5 to maximize the sample size, a minimum sample size of 385 participants was determined.

### 2.6. Questionnaire Tool

The study utilized a self-administered questionnaire available in English. It comprised 41 items divided into two main sections.

The first section (22 items) collected baseline information, including sociodemographic characteristics, lifestyle factors, physical characteristics, and dietary behaviors. Sociodemographic characteristics included (age, sex, marital status, nationality, residential setting, academic faculty, academic level, and year of study). Lifestyle factors included (alcohol and coffee consumption, smoking status, perceived stress levels, physical activity, and napping habits). The participants also self-reported their height and weight. Dietary behaviors assessed usual mealtime regularity, breakfast skipping, consumption of late-night snacks (defined as after 10 p.m.), substituting snacks for main meals, tendency to consume heavier evening meals, and the typical time interval between the last meal and bedtime.

The second section (19 items) consisted of the validated tool Pittsburgh Sleep Quality Index (PSQI), which was used to assess overall sleep quality and its components [16]. Dietary habit questions and categorization methods for sleep components were informed by and adapted from previous research by Faris et al. (2022), as discussed in the following sections [40]. A detailed classification of all questionnaire items is provided in Supplemental File S1.

The draft questionnaire underwent a pilot test with a small sample (*n* = 20) of international students to check the clarity and comprehensibility of the dietary and lifestyle practice questions. Minor changes were made to the wording of some questions. The internal consistency of the Pittsburgh Sleep Quality Index in this sample was acceptable (Cronbach’s α = 0.76).

#### 2.6.1. Anthropometric Assessment

Students self-reported their height (cm) and weight (kg), from which Body Mass Index (BMI) was calculated and categorized according to the standard classifications: underweight (BMI < 18.5 kg/m^2^), normal weight (BMI: 18.5–24.9 kg/m^2^), overweight (BMI: 25–29.9 kg/m^2^), obesity class I (BMI: 30–34.9 kg/m^2^), obesity class II (BMI: 35–39.9 kg/m^2^), and obesity class III (BMI > 40 kg/m^2^) [41].

Given the known differences in body composition, specific BMI classifications were applied to Asian students: underweight (BMI < 18.5 kg/m^2^), normal weight (BMI: 18.5–22.9 kg/m^2^), overweight (BMI: 23–24.9 kg/m^2^), obesity class I (BMI: 25–29.9 kg/m^2^), obesity class II (BMI: 30–34.9 kg/m^2^), and obesity class III (BMI ≥ 35 kg/m^2^) [42,43].

#### 2.6.2. Sleep Quality Assessment

The Pittsburgh Sleep Quality Index (PSQI) was used to assess participants’ sleep quality and patterns over the preceding month. The reliability and validity of the PSQI are well-established [16].

PSQI consists of 19 items categorized into seven parameters related to sleep, including sleep duration, sleep latency, subjective sleep quality, sleep disturbances, sleep efficiency, daytime dysfunction and use of sleeping medication.

The students rated these parameters on a scale from 0 to 3 individually, where 0 represented the best quality of sleep and 3 the worst quality of sleep. The individual component scores were then summed to yield a global PSQI score of 0 to 21. Higher global scores indicate worse sleep quality; 0 represents no sleep problems, whereas 21 indicates severe problems in all sleep components [16].

#### 2.6.3. Eating Habits Assessment

Respondents answered a set of questions about their eating behaviors in the previous month. These questions included inquiries about frequent breakfast skipping, late-night snack consumption (snack intake after 10:00 p.m.), and substituting snacks for main meals (breakfast, lunch, and dinner). Students were also asked about eating heavier evening meals (in terms of caloric density) and meal timing regularity (having main meals at the same time regularly). To elucidate, participants were provided with a list of snacks, including calorically dense items such as chips, baked goods, sweets, processed meats, cheeses, and beverages, excluding diet drinks, water, tea, and plain coffee. Participants responded with either yes or no to the presented options.

In addition, participants were instructed to report the interval between their last meal of the day and bedtime, choosing from defined categories of either 0–2 h, or greater than or equal to 3 h.

#### 2.6.4. Coding and Categorization of Variables

Ordinal scores of the global PSQI (ranging from 0 to 21) and each sleep component (ranging from 0 to 3) were converted into categorical variables. The PSQI global score was dichotomized: a score greater than 5 was identified as poor overall quality of sleep, while a score of 5 or less as good overall quality of sleep [16].

The PSQI subcomponents scores were categorized as either adequate or inadequate. For subjective sleep quality, scores of 0 or 1 were classified as adequate (representing high or medium quality), and scores of 2 or 3 were considered inadequate (representing low or very low quality). Similarly, sleep latency was categorized as adequate for scores of 0 or 1 (indicating a duration of 30 min or less to fall asleep) and inadequate for scores of 2 or 3 (indicating a duration of more than 30 min). For sleep efficiency (defined as the % of actual time spent asleep in bed), an efficiency of 75% or more (scores of 0 or 1) was classified as adequate, while an efficiency of 74% or less (scores of 2 or 3) was deemed inadequate. Sleep duration was considered adequate for six hours or more (scores of 0 or 1) and inadequate for less than six hours (scores of 2 or 3).

Sleep disturbance (assessed by the frequency of various sleep-related problems such as waking up at night, breathing issues, or experiencing bad dreams that interfere with sleep quality) was categorized as adequate when the sum of scores was less than 10 out of 27 on the PSQI sleep disturbance component score (representing no or low levels of disturbance), and inadequate when the sum of scores was 10 or more (representing medium or high levels). The need for sleep medication was deemed adequate with no or low usage (scores of 0 or 1) if participants reported using it less than once per week, and inadequate with medium or high usage (scores of 2 or 3) if used once per week or more.

Daytime dysfunction was classified as adequate for no or low levels of dysfunction; scores of 0 or 1 (indicating a lack of or only a slight problem with remaining alert during daily activities and maintaining sufficient motivation to complete tasks), and inadequate for scores of 2 or 3 for medium or high levels (indicating that the problem was sometimes or very frequently an issue). Finally, the time interval between the last meal and bedtime was considered adequate if it was three hours or more, and inadequate if it was less than three hours [40].

### 2.7. Ethical Approval

Ethical approval for this study was obtained from the Institutional Review Board of the University of Pécs (No.9568-PTE 2023).

### 2.8. Statistical Analysis

Statistical analyses were conducted using SPSS software (version 27). Categorical variables were presented as frequencies and percentages, while quantitative data were summarized using means and standard deviations (SD). Descriptive statistics were generated to characterize sociodemographic data and overall sleep quality. Sleep components and sex were compared using Pearson Chi-squared test.

Cross-tabulation and assessment of the strength of association were conducted between sleep components and eating habits, with results presented as odds ratios (OR), 95% confidence intervals (CI), and *p*-values. Multivariate logistic regression analysis was performed to calculate the odds ratio (OR), 95% confidence intervals (CI), and *p*-value, thereby assessing the association of dietary habits with overall sleep quality (as measured by the PSQI). The results were adjusted for the following demographic and lifestyle factors: age, sex, marital status, residential setting, academic faculty, academic level, year of study, nationality, BMI, smoking, physical activity, stress level, napping frequency, alcohol and coffee consumption. Statistical significance was defined as a *p*-value < 0.05. The logistic regression model was tested for multicollinearity using the Variance Inflation Factor (VIF). All VIF values were below 5, indicating that multicollinearity was not a critical concern in the model.

### 2.9. Consideration of Potential Bias

Several potential biases were considered. The use of convenience sampling introduces the risk of selection bias, as students with a particular interest in health might have been more likely to participate in the study. To mitigate this, recruitment was conducted across diverse campuses and faculties.

The use of self-reported data for dietary habits and anthropometrics is subject to recall, social desirability, and measurement biases. The use of the validated PSQI instrument helped standardize the primary outcome and reduce measurement bias for sleep quality.

## 3. Results

A total of 385 students were included in the present study, drawn from a population of approximately 5000 international students. The participants’ sociodemographic profiles are summarized in Table 1. The majority of the students (68.8%) were females, and most students (71.7%) were between 18 and 24 years of age. These participants originated from one of four broad regions: the Middle East and North Africa (MENA), Europe/Americas, Central, Eastern and South Asia, and Sub-Saharan Africa. A significant proportion (82.3%) of the students were single. Regarding academic enrollment, 68.6% were in faculties related to medicine, health sciences, and pharmacy. Another 19.5% were enrolled in engineering and sciences faculties, with the remaining 11.9% in business, humanities, law, music, or visual arts.

The majority of the students (70.1%) were pursuing undergraduate degrees, while 15.6% were in master’s programs and 14.3% in doctoral programs. A large proportion (70.4%) were in their first or second year of study. In terms of residential status, 79.7% lived off-campus. Smoking was reported by a minority (13.8%), while regular alcohol consumption (54.8%) and coffee or caffeinated beverage intake (50.6%) were common. Regular physical activity was reported by a smaller group (39.7%). Regarding body weight, (60.3%) had a normal BMI, while (30.1%) were classified as overweight or obese. Severe stress was reported by one-quarter of participants (25.7%). Additionally, a considerable proportion of participants reported poor sleep quality (51.7%), and the majority indicated daily napping (73.8%).

Table 2 presents the significant differences in sleep quality components based on sex.

For example, males reported poor sleep efficiency more often than females (27.5% vs. 15.8%, *p* = 0.008). Conversely, females more frequently experienced daytime dysfunction compared to males (27.9% vs. 8.3%, *p* < 0.001)

Table 3 presents the associations between specific eating behaviors and the global Pittsburgh Sleep Quality Index (PSQI) score, as well as its components. The results revealed that several eating behaviors were significantly associated with poorer sleep outcomes among the students. Specifically, frequent breakfast skipping was associated with an increased likelihood of longer sleep latency (OR = 1.53, 95% CI (1.00, 2.33), *p* = 0.048), increased odds of sleep medication use (OR = 3.12, 95% CI (1.48, 6.57), *p* = 0.002), and daytime dysfunction (OR = 1.67, 95% CI (1.03, 2.72), *p* = 0.038) as compared to breakfast consumption. Similarly, late-night snacking (after 10:00 p.m.) showed greater odds of poor overall sleep quality (OR = 1.59, 95% CI (1.06, 2.38), *p* = 0.024), prolonged sleep latency (OR = 1.54, 95% CI (1.00, 2.35), *p* = 0.046), and reduced sleep efficiency (OR = 2.10, 95% CI (1.23, 3.58), *p* = 0.006) compared to no late-night snacking.

Students who substituted snacks for main meals demonstrated significant associations with adverse sleep outcomes, including increased odds of poor overall sleep quality (OR = 3.00, 95% CI (1.97, 4.55), *p* < 0.001), poor subjective sleep quality (OR = 2.07, 95% CI (1.31, 3.26), *p* = 0.002), prolonged sleep latency (OR = 2.64, 95% CI (1.72, 4.06), *p* < 0.001), reduced sleep efficiency (OR = 2.17, 95% CI (1.29, 3.63), *p* = 0.003), and shorter sleep duration (OR = 2.50, 95% CI (1.61, 3.87), *p* < 0.001). The likelihood of sleep medication use (OR = 4.50, 95% CI (1.99, 10.19), *p* < 0.001) and daytime dysfunction (OR = 2.15, 95% CI (1.31, 3.52), *p* = 0.002) was also substantially higher in this group compared to students who did not.

Irregular meal timing was linked with adverse sleep parameters, including poor subjective sleep quality (OR = 2.68, 95% CI (1.17, 6.15), *p* = 0.016) and prolonged sleep latency (OR = 2.59, 95% CI (1.26, 5.34), *p* = 0.008) compared to regular meal timing. Furthermore, students who habitually consumed heavier evening meals showed significantly increased odds of poor overall sleep quality (OR = 2.10, 95% CI (1.39, 3.15), *p* < 0.001), poor subjective sleep quality (OR = 1.65, 95% CI (1.04, 2.61), *p* = 0.033), reduced sleep efficiency (OR = 2.23, 95% CI (1.30, 3.83), *p* = 0.003), and increased sleep medication use (OR = 3.15, 95% CI (1.39, 7.13), *p* = 0.004) compared to students who did not.

Finally, consuming a meal within three hours before bedtime was associated with poor overall sleep quality (OR = 2.38, 95% CI (1.58, 3.61), *p* < 0.001), poor subjective sleep quality (OR = 2.50, 95% CI (1.58, 3.96), *p* < 0.001), prolonged sleep latency (OR = 2.02, 95% CI (1.32, 3.08), *p* = 0.001), reduced sleep duration (OR = 1.61, 95% CI (1.05, 2.48), *p* = 0.028), and diminished sleep efficiency (OR = 1.99, 95% CI (1.19, 3.31), *p* = 0.008). This eating behavior was also linked to higher odds of sleep medication use (OR = 2.07, 95% CI (1.02, 4.21), *p* = 0.041) and daytime dysfunction (OR = 2.14, 95% CI (1.31, 3.50), *p* = 0.002) compared to consuming the last meal more than three hours before bedtime.

Table 4 presents the relationship between dietary habits and overall sleep quality (measured by the Pittsburgh Sleep Quality Index, PSQI).

These analyses were conducted while statistically accounting for the influence of multiple demographic and lifestyle confounders, including age, sex, marital status, nationality, residential setting, academic level, academic faculty, year of study, caffeine intake, smoking status, alcohol consumption, physical activity, stress levels, napping habits, and body mass index. Multiple logistic regression analysis identified heavy evening meals (OR = 2.73, 95% CI (1.58, 4.73), *p* < 0.001), substituting snacks for main meals (OR = 2.68, 95% CI (1.47, 4.90), *p* = 0.001), and consuming the last meal within three hours before bedtime (OR = 2.06, 95% CI (1.17, 3.63), *p* = 0.012) as the strongest factors associated with poor sleep quality.

**Figure 1:**
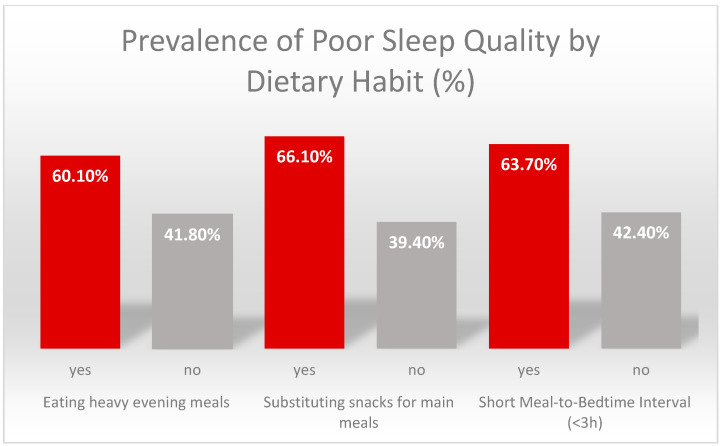
Prevalence of Poor Sleep Quality by Dietary Habit. The figure outlines the percentage of students with poor sleep quality (Global PSQI > 5) among those who reported engaging in specific unhealthy dietary behaviors versus those who did not. After controlling for key demographic and lifestyle confounders, the adjusted odds ratios (aOR) for these associations were: short meal to bedtime interval, aOR = 2.06, replacing meals with snacks, aOR = 2.68, and heavy evening meals, aOR = 2.73.

The strong associations between these key dietary habits and poor sleep quality are summarized in Figure 1.

## 4. Discussion

This study investigated the association of specific unhealthy dietary behaviors with sleep quality among international university students in Pécs, Hungary. According to the findings, over half of the students surveyed experienced poor sleep quality. The high rate of poor sleep quality found in this sample reflects the stressful university environment and the accompanying lifestyle changes students face during this period [7,8,9,10]. For international students, cultural adaptation, language barriers, and isolation from support systems pose additional stressors.

Notably, sex-based variations in sleep quality were observed, with female students reporting poorer sleep quality than male students. This is consistent with prior research in Australia (65.1% vs. 49.8%), New Zealand (63.1% vs. 44.5%), and Spain (55.7% vs. 37.0%), where female university students reported poorer overall sleep quality than males [44,45,46]. Biological factors are extremely important; hormonal alterations throughout the menstrual cycle might strongly impact sleep architecture by their effect on core body temperature and the regulation of sleep-promoting hormones, such as melatonin [47]. Psychosocial factors also have a strong effect, as young adult women regularly report higher levels of perceived depression, stress, and anxiety, possibly affecting cognitive hyperarousal and rumination prior to sleep, which could ultimately affect sleep onset and sleep maintenance [48]. Furthermore, lifestyle and social factors might be implicated, as women have a greater load of emotional and household labor, which would constrain their opportunities to have restorative and deep sleep [49,50]. Hence, the observed differences are most likely due to the interplay of physiological, social, and psychological factors [51].

Differences in sleep parameters depending on sex were also evident, with male students having lower sleep efficiency and female students having significantly higher levels of daytime dysfunction. Targeted strategies are needed to address the unique sleep quality variations across both groups.

The results further identified a strong association between poor sleep quality and its parameters (such as daytime dysfunction, sleep duration, efficiency, latency, disturbance, subjective sleep quality, and sleep medication usage) and key unhealthy dietary patterns. These dietary habits included irregular meal timing, substituting snacks for main meals, late-night snacking, consuming heavy evening meals, breakfast skipping, and short meal-to-bedtime intervals.

### 4.1. Mealtime and Sleep Quality

The findings of this study add to the mounting evidence demonstrating a robust link between chrono-nutrition and sleep quality by identifying three independent dietary factors associated with poor sleep quality: short interval between the last meal and bedtime, substituting snacks for main meals, and heavy evening meals. The association of these dietary habits with poor sleep quality remained statistically significant after controlling for potential confounders, indicating a strong and independent association with poor sleep outcomes.

#### 4.1.1. Short Meal-to-Bedtime Interval (<3 h)

Having a meal within three hours of bedtime was independently and significantly associated with poorer sleep quality, which is consistent with previous research illustrating negative associations of excessive food intake prior to bedtime with several aspects of sleep quality [52,53].

In addition to directly disturbing sleep processes, eating shortly before sleep time may elevate core body temperature, which could subsequently evoke a phase delay in the circadian rhythm, which can impact the timing and quality of sleep onset and maintenance [30]. Furthermore, consuming food within a short time frame prior to sleep is associated with gastrointestinal problems, most significantly gastroesophageal reflux disease (GERD), eventually influencing sleep quality.

In a case–controlcase-control study, participants with a dinner-to-bed interval of less than three hours had an increased odds ratio of 7.45 for GERD compared to those with at least a four-hour dinner-to-bed interval [54]. A recent systematic review and a cross-sectional study support this finding, confirming that a short meal-to-sleep interval is a significant risk factor for GERD. Overall, this suggests a minimum three-hour digestion window before sleep onset [55,56].

#### 4.1.2. Substituting Snacks for Main Meals

Substituting main meals for snacks had a robust association with sleep quality. This is in line with a previous cross-sectional survey of 498 university students, which reported a positive association between substituting snacks for main meals and a higher risk of poor sleep quality [40]. This can be explained by the generally high energy density of snacks; previous studies in animals and humans have found that excess caloric consumption, specifically from fats, negatively affects circadian gene expression, consequently worsening sleep quality outcomes [57].

A study conducted on American women aged 35 to 74 years suggested that greater reliance on substituting snacks for meals and a lower tendency to eat at conventional mealtimes were associated with greater energy intake from fat and sweets and lower intake of fruits and vegetables [58]. Such eating patterns may result in deficiencies in several key sleep regulation nutrients, including magnesium, tryptophan, and zinc [59]. Another European study conducted in Denmark exhibited a direct correlation between the frequency of energy-dense sugary snacks consumption and poor sleep outcomes (e.g., short sleep duration and irregular sleep patterns) [60].

#### 4.1.3. Heavy Evening Meals

Consuming heavy evening meals was significantly associated with worse sleep quality, consistent with earlier studies; for example, Charlotte et al. (2024) demonstrated an association between greater evening food intake and worse sleep quality [61].

The timing of food consumption acts as an important zeitgeber for our complex circadian rhythms; consuming heavy evening meals can lead to desynchronization between the central pacemaker and peripheral clocks, consequently disturbing metabolic processes and the sleep-wake cycle [31].

### 4.2. Other Dietary Habits and Their Associations with Sleep Components

Certain dietary behaviors were not identified as statistically significant association factors with overall poor sleep quality in the adjusted model; however, they demonstrated some meaningful associations with individual sleep parameters in the cross-tabulations analysis.

#### 4.2.1. Skipping Breakfast

Breakfast skipping did not demonstrate a significant independent correlation with overall poor sleep in the multivariate analysis; however, it showed associations with multiple adverse sleep outcomes. In particular, the results suggested that breakfast skipping correlates with increased sleep medication usage, higher levels of daytime dysfunction, and longer sleep onset latency. These results are in line with those reported in the literature. Researchers have regularly found an association between breakfast skipping and poor quality of sleep components among university students [62]. Similarly, a randomized crossover design study found that participants of university students had shorter sleep latency with breakfast consumption compared to longer sleep latency with breakfast skipping [63].

Daytime dysfunction has been characterized by altered mood, reduced cognitive functioning, energy, and overall productivity. In line with this, a review demonstrated associations between skipping breakfast and mood swings and midday fatigue, likely due to a drop in blood sugar [64], which corroborates our findings. Furthermore, college students who skipped breakfast had significantly lower scores on cognitive tests evaluating attentiveness, reaction time, alertness and overall performance than individuals who regularly consumed breakfast [65]. In another intervention study, researchers observed that those who regularly consumed breakfast experienced reduced daytime dysfunction and improved sleep quality compared to those who regularly skipped breakfast [66].

Although there is insufficient research documenting a direct causal relationship between breakfast omission and the use of sleep medications, chronic sleep problems due to skipping breakfast may coincide with seeking sleep aids, thereby increasing the likelihood of using sleep medications.

Breakfast skipping is defined as the intentional or unintentional omission of breakfast at least once a week. Despite the well-established benefits of regular breakfast consumption, the high prevalence of skipping this meal remains a significant public health concern. Estimates have shown that breakfast skipping among young children and adolescents globally is 10% to 30%, with this figure rising dramatically to over 50% among university students; for instance, in Bangladesh, 52.8% of university students reported skipping breakfast, and those with poor sleep quality were three times more likely to skip breakfast than their peers. Additionally, female university students were more likely to skip breakfast than male university students, a trend potentially linked to concerns about body image and weight control [67,68].

#### 4.2.2. Late-Night Snacking (After 10:00 p.m.)

Although the multivariate model did not identify late-night snacking as an independent factor associated with poor sleep, cross-tabulation analysis demonstrated an association between late-night snacking and increased sleep latency and reduced sleep efficiency. Overall, these results support past research suggesting that consuming food, including snacks late at night (after 10:00 p.m.), is associated with a greater risk of compromised sleep quality [40].

The mechanisms underpinning this association are likely multifactorial. University students often consume snacks such as chips, sweets, and confectionery [69]. These highly processed, energy-dense foods are easily overconsumed, leading to increased overall caloric and fat intake. This dietary pattern has been directly linked to poorer sleep outcomes. For example, a clinical study demonstrated a negative association between the consumption of saturated, polyunsaturated, trans, and total fats and sleep duration [70]. Another study conducted by Carvalho et al. (2020) among night-shift workers revealed that pre-sleep food intake with high fat and carbohydrate content increased sleep onset latency [71]. Conversely, Bravo et al. (2012) found that a special pre-sleep snack, such as cereals with high tryptophan content, would shorten sleep latency, which contradicts our results [72]. This indicates the importance of both the type and timing of pre-sleep food consumption in determining sleep outcomes.

Late-night snack consumption could also indirectly affect sleep outcomes by changing the subsequent food consumption schedule, for example, increasing the likelihood of skipping breakfast, which has been shown previously to correlate with poorer sleep quality [73,74,75]. In a trial of sixteen adults, sleep restriction resulted in a statistically significant increase in caloric intake (42% increase) from post-dinner, carbohydrate, protein, and fiber-rich snack consumption [76]. This suggests a bidirectional relationship where poor sleep could drive late-night snack consumption, and vice versa.

#### 4.2.3. Irregular Meal Timing

Regression analysis did not demonstrate statistical significance for irregular meal timing. Yet, the consistency of the observed associations with sleep parameters warrants additional investigation. Irregular meal schedules were associated with prolonged sleep latency and diminished subjective sleep quality, likely due to the impact of regular mealtime on the circadian rhythm synchronization [77].

Irregular mealtime patterns can disrupt metabolic regulation, causing circadian misalignment [78]. Supporting this, Hatori et al. (2012) revealed that irregular feeding patterns dysregulate circadian rhythms, while regular feeding schedules help entrain them [79]. Later research also showed a significant association between irregular meal patterns and poorer sleep quality [80]. Similarly, a cross-sectional study reported a strong relationship between irregular mealtimes and longer sleep latency [40].

A key strength of this study is the use of a well-validated tool that evaluates several sleep parameters (PSQI) [16]. Additionally, the sample size used in this study; the study recruited a large sample size of students reinforcing the statistical power regarding the prevalence of poor sleep quality. Furthermore, targeting international students, a demographic that frequently experiences extreme changes in lifestyle and eating patterns, provides strong insights into the sleep problems facing this vulnerable population. Finally, the study considered several potential lifestyle and demographic confounding variables in the multivariate regression analysis, thereby enhancing the validity of the findings.

However, several limitations should be acknowledged. A key weakness of this study is its cross-sectional methodology, which allowed for exploration of the associations between unhealthy eating behaviors and poor sleep quality, but could not establish causality. Additionally, the restricted inclusion of students using a convenience sample from a single university might be susceptible to self-selection bias and compromise the external validity of the findings. Furthermore, dietary patterns assessment relied on self-reported, non-validated questions, introducing the potential for measurement bias. Moreover, although we controlled for several lifestyle factors, these were measured with broad categories (e.g., regular/irregular physical activity/coffee drinker), and the lack of data on intensity, duration, or precise timing may have limited our ability to fully account for their confounding effects. Finally, this research did not account for several established confounders in chrono-nutrition research, including individual chronotype, diet type, detailed nutrient composition and caloric intake, and data on environmental factors, such as light and noise, and mental health status were not collected.

Future longitudinal studies with larger sample sizes should incorporate objective measures, detailed dietary assessment, and consider confounding variables to confirm these results and investigate the influence of culture and age on the diet-sleep relationship, providing a more comprehensive assessment of factors influencing sleep quality outcomes

### 4.3. Practical Implications and Future Directions

First: enhance university health services; health centers should systematically screen for sleep problems and provide access to dietitians and doctors for personalized consultations on the identified risk factors. Second: develop accessible digital educational tools; create mobile applications to provide support and education on healthy eating behavior and sleep hygiene. In addition, include principles of chrono-nutrition in university workshops and health promotion programs, advising students to limit heavy evening meals and maintain a meal-to-bedtime interval of at least three hours. Third: restructure the campus environment; provide healthier, appealing snacks and meals in vending machines and cafeterias to reduce the substitution of energy-dense, nutrient-poor snacks for main meals, and provide healthy beverages as affordable alternatives to sugary drinks and high-caffeine energy drinks. Fourth: provide practical skill-building support; offer dorm-friendly meal and snack preparation workshops to teach simple, healthy meal and snack preparation skills to reduce reliance on convenience, unhealthy snacks. Finally: combine sleep hygiene education with time-management workshops to help students organize their schedules for consistent meals and sufficient sleep.

## 5. Conclusions

Unhealthy dietary habits, particularly consuming heavy evening meals, substituting snacks for main meals, and a short meal-to-bedtime interval, are significantly associated with poor sleep quality among international university students. These results demonstrate the important relationship between chrono-nutrition and sleep health in this population.

Interventions promoting appropriate meal timing and healthier eating patterns as part of comprehensive health promotion strategies could be an effective strategy to improve sleep quality in this population.

## Figures and Tables

**Table 1:** General characteristics and sleep quality of the students (*n* = 385).

Characteristics	Frequency (*n*)	Percentage (%)
**Sex**		
Male	120	31.2
Female	265	68.8
**Age group**		
18–24	276	71.7
25–30	75	19.5
30–40	34	8.8
**Year of study**		
1st and 2nd Year	271	70.4
3rd and 4th year	100	26.0
5th and 6th year	14	3.6
**Academic faculty**		
Faculty of Engineering and Sciences	86	19.5
Faculty of Medicine, Health Sciences and Pharmacy	265	68.6
Faculty of Business, Cultural Sciences, Humanities, Law, Music and Visual Arts	34	11.9
**Academic level**		
Bachelor	270	70.1
Master	60	15.6
PhD	55	14.3
**Marital status**		
Single	317	82.3
Married	16	4.2
Other	52	13.5
**Residential setting**		
Dormitory	78	20.3
Apartment	307	79.7
**Smoking status**		
Smoker	53	13.8
Non-smoker	332	86.2
**Alcohol consumption**		
Irregular alcohol drinker	174	45.2
Regular alcohol drinker	211	54.8
**Coffee consumption**		
Irregular coffee drinker	190	49.4
Regular coffee drinker	195	50.6
**Physical activity**		
Irregular physical activity exerciser	232	60.3
Regular physical activity exerciser	153	39.7
**Stress level**		
Normal	78	20.3
Mild	71	18.4
Moderate	137	35.6
Severe	99	25.7
**Napping frequency**		
Do not nap	90	23.3
Once a day	284	73.8
More than once	11	2.9
**Nationality**		
European and Americans	110	28.6
Middle Eastern and North African	134	34.8
Sub-Saharan African	52	13.5
Central, Eastern and South Asia	89	23.1
**BMI (kg/m^2^)**		
Underweight	37	9.6
Normal weight	232	60.3
Overweight & Obese class 1, 2, and 3	116	30.1
**Global sleep quality (PSQI)**		
Poor sleep quality	199	51.7
Good sleep quality	186	48.3

BMI, body mass index; PSQI, Pittsburgh Sleep Quality Index.

**Table 2:** Comparison of sleep quality and its components between male and female students.

Sleep Behaviors	Male (*n* = 120)	Female (*n* = 265)	Total (*n* = 385)	X^2^	*p*-Value
	*n* (%)	*n* (%)	*n* (%)		
**Subjective sleep quality**					
Adequate subjective sleep quality ^1^	88 (73.3)	192 (72.5)	280 (72.7)	0.32	0.857
Inadequate subjective sleep quality ^2^	32 (26.7)	73 (27.5)	105 (27.3)		
**Sleep latency**					
Adequate sleep latency ^1^	79 (65.8)	169 (63.8)	248 (64.4)	0.153	0.696
Inadequate sleep latency ^2^	41 (34.2)	96 (36.2)	137 (35.6)		
**Sleep Duration**					
Adequate sleep latency ^1^	83 (69.2)	176 (66.4)	259 (67.3)	0.284	0.594
Inadequate sleep latency ^2^	37 (30.8)	89 (33.6)	126 (32.7)		
**Sleep efficiency**					
Adequate sleep efficiency ^1^	87 (72.5)	223 (84.2)	310 (80.5)	7.148	0.008 *
Inadequate sleep efficiency ^2^	33 (27.5)	42 (15.8)	75 (19.5)		
**Sleep disturbance**					
Adequate sleep disturbance ^1^	106 (88.3)	224 (84.5)	330 (85.7)	0.977	0.323
Inadequate sleep disturbance ^2^	14 (11.7)	41 (15.5)	55 (14.3)		
**Use of Sleep Medication**					
Adequate use of sleep Medication ^1^	113 (94.2)	237 (89.4)	350 (90.9)	2.239	0.135
Inadequate use of sleep Medication ^2^	7 (5.8)	28 (10.6)	35 (9.1)		
**Daytime dysfunction**					
Adequate daytime dysfunction ^1^	110 (91.7)	191 (72.1)	301 (78.2)	18.585	<0.001 *
Inadequate daytime dysfunction ^2^	10 (8.3)	74 (27.9)	84 (21.8)		
**Global PSQI Score**					
Global score ≤ 5 (good overall sleep quality) ^3^	66 (55.0)	120 (45.3)	186 (48.3)	3.123	0.077
Global score > 5 (poor overall sleep quality) ^4^	54 (45.0)	145 (54.7)	199 (51.7)		

* The *p*-value was obtained from Pearson’s chi-square (sig. 2-sided), statistically significant at *p* < 0.05. ^1^ Adequate indicates relevant sleep component scores (0 or 1) on the Pittsburgh Sleep Quality Index (PSQI). ^2^ Inadequate indicates relevant sleep component scores (2 or 3) on the Pittsburgh Sleep Quality Index (PSQI). ^3^ A global score of ≤5 indicates good overall sleep quality according to the Pittsburgh Sleep Quality Index (PSQI). ^4^ A global score of >5 indicates poor overall sleep quality according to the Pittsburgh Sleep Quality Index (PSQI).

**Table 3:** Cross-tabulation and association measures of eating habit factors with poor overall sleep and its components (OR, and 95% CI).

Eating Habits	Overall Sleep Quality	Subjective Sleep Quality	Sleep Latency	Sleep Duration	Sleep Efficiency	Sleep Disturbances	Use Sleep Medication	Daytime Dysfunction
**Skipping breakfast**	1.24 (0.83–1.86)	1.46 (0.93–2.30)	**1.53 (1.00–2.33)**	1.47 (0.96–2.25)	1.33 (0.80–2.21)	1.67 (0.94–2.96)	**3.12 (1.48–6.57)**	**1.67 (1.03–2.72)**
**Late-night snacking**	**1.59 (1.06–2.38)**	1.58 (1.00–2.50)	**1.54 (1.00–2.35)**	1.35 (0.88–2.07)	**2.10 (1.23–3.58)**	1.61 (0.89–2.90)	0.79 (0.40–1.59)	1.53 (0.93–2.51)
**Substituting snacks for main meals**	**3.00 (1.97–4.55)**	**2.07 (1.31–3.26)**	**2.64 (1.72–4.06)**	**2.50 (1.61–3.87)**	**2.17 (1.29–3.63)**	1.63 (0.92–2.89)	**4.50 (1.99–10.19)**	**2.15 (1.31–3.52)**
**Eating heavy evening meals**	**2.10 (1.39–3.15)**	**1.65 (1.04–2.61)**	1.20 (0.79–1.83)	0.75 (0.49–1.15)	**2.23 (1.30–3.83)**	1.03 (0.58–1.82)	**3.15 (1.39–7.13)**	1.42 (0.87–2.32)
**Irregular mealtime**	1.00 (0.55–1.78)	**2.68 (1.17–6.15)**	**2.59 (1.26–5.34)**	1.54 (0.79–3.00)	1.02 (0.49–2.14)	1.66 (0.63–4.38)	1.74 (0.51–5.89)	1.39 (0.65–2.98)
**Short Meal-to-Bedtime Interval (<3 h)**	**2.38 (1.58–3.61)**	**2.50 (1.58–3.96)**	**2.02 (1.32–3.08)**	**1.61 (1.05–2.48)**	**1.99 (1.19–3.31)**	1.67 (0.94–2.96)	**2.07 (1.02–4.21)**	**2.14 (1.31–3.50)**

This table measures the association between specific dietary habits and the Pittsburgh Sleep Quality Index (PSQI) parameters. Overall sleep quality categorized into: poor overall sleep quality (Global PSQI score > 5), and good overall sleep quality (Global PSQI score ≤ 5) [8]. Other sleep parameters were classified into two outcomes and analyzed as a categorical variable: adequate (component score 0 or 1) and inadequate (component score 2 or 3). Values presented in **bold** indicate statistical significance at a *p*-value less than 0.05. Risk assessment and crosstabs were conducted to calculate the odds ratio (OR) and its 95% confidence interval (CI), representing the strength of these associations.

**Table 4:** Adjusted multiple logistic regression showing eating habits associated with overall poor sleep quality.

Eating Habit	Odds Ratio	95% CI ()	*p*-Value
Substituting snacks for main meals	**2.68**	(1.47–4.90)	**0.001**
Heavy evening meals	**2.73**	(1.58–4.73)	**<0.001**
Short Meal-to-Bedtime Interval (<3 h)	**2.06**	(1.17–3.63)	**0.012**
Irregular mealtime	0.58	(0.27–1.26)	0.168
Skipping breakfast	0.67	(0.39–1.15)	0.144
Late-night snacking	0.82	(0.44–1.51)	0.517

Multiple logistic regression showing dietary habits that are associated with poor sleep quality among university students, adjusted for sociodemographic and lifestyle factors (age, sex, marital status, residential setting, academic faculty, year of study, nationality, BMI, academic level, smoking, alcohol and coffee consumption, physical activity, stress level and napping frequency). CI, confidence interval. Values presented in bold indicate statistical significance at a *p*-value less than 0.05 (*p* < 0.05).

## Data Availability

The data presented in this study are available upon request to the corresponding author.

## Supplementary Materials

The following supporting information can be downloaded at: https://www.mdpi.com/article/10.3390/nu17223580/s1, Supplementary Material File S1; The self-administered questionnaire used for data collection, including sociodemographic, dietary behavior, and Pittsburgh Sleep Quality Index (PSQI) sections.

## Abbreviations

The following abbreviations are used in this manuscript:

SQ	Sleep Quality
PSQI	Pittsburgh Sleep Quality Index
OR	Odds Ratio
MENA	Middle Eastern & North African
AHI	Apnea-Hypopnea Index
STROBE	Strengthening the reporting of observational studies in epidemiology
WHO	World Health Organisation
P	Population Proportion
GERD	Gastroesophageal Reflux Disease
