# Supplementary material for: The Association of Unhealthy Eating Behaviors with Sleep Quality Outcomes Among University Students: A Cross-Sectional Study
Source: Nutrients. 2025 Nov 15;17(22):3580. doi: 10.3390/nu17223580 (PMC12655729; doi:10.3390/nu17223580)
Supplement: Supplementary file 1 [file nutrients-17-03580-s001.zip › nutrients-3937369-supplementary.pdf]

Baseline Questionnaire

1. Age:………………….

2. Sex

(1) Male
(2) Female

3. What degree are you studying?

(1) Bachelor
(2) Master
(3) PhD

4. In which year?

(1) First year
(2) Second
(3) Third
(4) Fourth
(5) Fifth
(6) Sixth

5. What is your faculty?

(1) Faculty of Business and Economics
(2) Faculty of Cultural Sciences, Education and Regional Development
(3) Faculty of Engineering and Information Technology
(4) Faculty of Health Sciences
(5) Faculty of Humanities and Social Sciences
(6) Faculty of Law
(7) Faculty of Music and Visual Arts
(8) Faculty of Pharmacy
(9) Faculty of Sciences
(10) Medical School

6. Your height ……….......cm

7. Your weight ....kg

8. Marital status?

(1) Single
(2) Married
(3) Other

9. Your stay in Hungary?

(1) Dormitory
(2) Apartment

10. What is your nationality?

………………………..

11. Current smoking status?

(1) Smoker
(2) Non-smoker

12. How often do you drink alcohol?

(1) Never
(2) Occasionally
(3) 1-2 times a week
(4) 3-5 times a week
(5) More than 5 times a week

13. How much coffee or other caffeinated beverage (e.g, energy drinks) do you drink?

(1) Never
(2) Occasionally
(1) 1-2 times a week
(2) 3-5 times a week
(3) More than 5 times a week

14. How would you describe your physical activity?

(1) I don't exercise
(2) I exercise once a week
(3) I exercise twice a week
(4) I exercise three or more times a week

15. <u>Life Stress</u>: How would you describe your stress level in your everyday life?

(1) Normal
(2) Mild
(3) Moderate
(4) Severe

16. How many times do you usually nap during the day?

(1) 0
(2) 1
(3) More than once

Instructions: The following questions relate to your usual dietary habits during the past month. Your answers should indicate the most accurate reply for the majority of days and nights in the past month

17. Do you usually have your meals at regular times? meals like breakfast/lunch/dinner.

(1) No
(2) Yes

18. Do you usually skip breakfast?

(1) Yes
(2) No

19. Do you usually have late-night snacks (after 10 pm)?

(e.g, chips, sweets, baked items, convenience foods like processed meat and cheese, and all beverages except diet drinks, water, plain tea and coffee).

(1) Yes
(2) No

20. Do you usually replace meals with snacks? meals like breakfast/lunch/dinner.

(1) Yes
(2) No

21. **Do you usually eat heavier meals in the evening compared to the daytime?**
(1) Yes
(2) No

22. **When do you usually have your last meal?**
(1) 0-2 hours before bedtime
(2) ≥3 hours before bedtime

Pittsburgh Sleep Quality Index
(PSQI)

Instructions: The following questions relate to your usual sleep habits during the past month only. Your answers should indicate the most accurate reply for the majority of days and nights in the past month. **Please answer all questions.**

1. During the past month, what time have you usually gone to bed at night? ______________________________

2. During the past month, how long (in minutes) has it usually taken you to fall asleep each night?

____

3. During the past month, what time have you usually gotten up in the morning?

____

4. During the past month, how many hours of actual sleep did you get at night? (This may be different than the number of hours you spent in bed.) __________________________

5. During the past month, how often have you had trouble sleeping because you…	Not during the past month	Less than once a week	Once or twice a week	Three or more times a week
a. Cannot get to sleep within 30 minutes				
b. Wake up in the middle of the night or early morning				
c. Have to get up to use the bathroom				
d. Cannot breathe comfortably				
e. Cough or snore loudly				
f. Feel too cold				

g. Feel too hot				
h. Have bad dreams				
i. Have pain				
j. Other reason(s), please describe:				
6. During the past month, how often have you taken medicine to help you sleep (prescribed or "over the counter")?				
7. During the past month, how often have you had trouble staying awake while driving, eating meals, or engaging in social activity?				
	No problem at all	Only a very slight problem	Somewhat of a problem	A very big problem
8. During the past month, how much of a problem has it been for you to keep up enough enthusiasm to get things done?				
	Very good	Fairly good	Fairly bad	Very bad
9. During the past month, how would you rate your sleep quality overall?				

	No bed partner or room mate	Partner/room mate in other room	Partner in same room but not same bed	Partner in same bed
10. Do you have a bed partner or room mate?				
	Not during the past month	Less than once a week	Once or twice a week	Three or more times a week
If you have a room mate or bed partner, ask him/her how often in the past month you have had:				
a. Loud snoring				
b. Long pauses between breaths while asleep				
c. Legs twitching or jerking while you sleep				
d. Episodes of disorientation or confusion during sleep				
e. Other restlessness while you sleep, please describe:				
